# Flow cytometric evaluation of CD4^+^ and CD8^+^ T-cell in IPB-D2 chickens with different Newcastle disease antibody titers level

**DOI:** 10.14202/vetworld.2023.1161-1164

**Published:** 2023-05-30

**Authors:** Dwi Lestari, Sri Murtini, Niken Ulupi, Asep Gunawan, Cece Sumantri

**Affiliations:** 1 Graduate School of Animal Production and Technology, Faculty of Animal Science, IPB University, Bogor, Indonesia; 2 Department of Animal Production and Technology, Faculty of Animal Science, IPB University, Bogor, Indonesia; 3 Department of Animal Disease and Veterinary Public Health, School of Veterinary Medicine and Biomedical Science, IPB University, Bogor, Indonesia

**Keywords:** cluster of differentiation 4^+^, cluster of differentiation 8^+^, flow cytometry, IPB-D2 chicken, lymphocytes

## Abstract

**Background and Aim:**

IPB-D2 chickens are selected from IPB-D1 due to their disease-resistance characteristics. One-way to evaluate the strength of a chicken’s immune system is by examining the number of circulating T lymphocytes. This assessment can be conducted using a modern analytic method called flow cytometry which relies on monoclonal antibodies to detect the relative proportions of each cell and measure the quality and quantity of biological and physical features of cells, including specific membrane or intracellular glycoprotein markers. Therefore, this study aimed to evaluate the population of lymphocytes, cluster of differentiation (CD)4^+^ and CD8^+^ in IPB-D2 chickens.

**Materials and Methods:**

Flow cytometry was used to evaluate the population of lymphocytes, CD4^+^, and CD8^+^ in IPB-D2 chickens. The data obtained in this study were analyzed by Minitab, and the mean values were compared using a t-test.

**Results:**

The lymphocytes, CD4^+^, and CD8^+^ populations of IPB-D2 chicken with high Newcastle disease (ND) antibody titers were 65.04%, 10.53%, and 5.47%. Meanwhile, this breed, with low ND antibody titers had lymphocytes CD4^+^ and CD8^+^ population of 57.19%, 8.40%, and 4.11 %. The comparison of CD4^+^ and CD8^+^ populations in chickens with high and low ND antibody titers was 1.92 and 2.04, respectively.

**Conclusion:**

IPB-D2 chickens with high ND antibody titers exhibited increased lymphocyte, CD4^+^, and CD8^+^ cell populations in comparison to those with low ND antibody titers. However, the high ND antibody titer group had a lower CD4^+^/CD8^+^ ratio.

## Introduction

The demand for local chicken in Indonesia has increased significantly as the lifestyle and preferences of people have evolved. However, local chicken farming poses the risk of disease incidence and mortality. Poultry production is a complicated system influenced by management, disease agents, and the environment. Despite efforts to address disease problems in local chickens farm, they continue to be a significant challenge that can result in substantial economic losses. To combat this, IPB-D2 chickens have been developed as an Indonesian breed selected based on their disease-resistant traits [1]. They are selected from IPB-D1 based on immunocompetence such as immunoglobulin Y concentration ≥10 mg/mL and Newcastle disease (ND) antibody titer ≥3 log_2_ HI unit.

Immunocompetence refers to the ability of an individual to mount an effective immune response against a pathogen [2], which can be evaluated by several parameters including circulating T lymphocyte populations. The amount and proportion of T cell subsets in circulation and organs have been correlated with disease susceptibility [3]. T lymphocytes play a key role in activating the immune system in response to specific diseases and stressors [4]. These cells are grouped into cluster of differentiation (CD)4^+^ and CD8^+^. This is because CD4 and CD8 bind major histocompatibility complex (MHC) Class II and MHC Class I molecules, respectively. T helper (Th) cells, also known as CD4^+^ T cells, produce cytokines and co-stimulatory molecules that aid other immune cells, such as B cells and innate immunity cells. On the other hand, CD8^+^ cells produce cytotoxic molecules such as perforin that can eliminate host cells infected by bacteria [5]. Cluster of differentiation molecules are surface indicators that determine the type of cell, stage of differentiation, and activity. These markers are recognized by particular sets of antibodies. CD4^+^ is located on Th cells, regulatory T-cells, monocytes, macrophages, and dendritic cells. Meanwhile, CD8^+^ is expressed on the surface of cytotoxic T-cells and natural killer cells [6].

Flow cytometry analysis of avian immune cells is an important and promising technique that enables studies on immunological competence and function in significant livestock species such as chicken or Turkey [7].

In layer chickens, the average CD4 and CD8 populations were highest at day one and lowest at twenty weeks of age [3]. According to Lee [8], the gut microbiota controls CD4^+^CD8^-^CD25^+^ and CD4^+^CD8^+^CD25^+^ population and function. T cells in the cecal tonsils and acetate can be crucial for maintaining the balance of the gut immune system. However, there are no investigations about the lymphocyte population in Indonesian local chickens, particularly the IPB-D2 breed. Therefore, this study aimed to evaluate the lymphocyte, CD4^+^, and CD8^+^ populations in IPB-D2 chickens.

## Materials and Methods

### Ethical approval

This study and all the tests and procedures were approved by the Institutional Animal Care and Use Committee (IACUC) at IPB University (approval number: 224-2021).

### Study period and location

This study was conducted between October and December 2021, and the maintenance of IPB-D2 chicken was performed in the field laboratory of The Faculty of Animal Science at IPB University. The flow cytometry analysis was conducted at the Mochtar Riady Institute for Nanotechnology, Universitas Pelita Harapan, Tangerang-Banten, Indonesia.

### Animals and blood collection

The samples used in this study were 8 IPB-D2 chickens aged 21 weeks. They were divided into high (3.25 ± 0.5 log_2_ HI unit) and low (1.00 ± 1.15 log_2_ HI unit) ND antibody titers. The blood sample was collected from the vena brachialis using a 3 mL syringe and stored in a cool box for further analysis.

The chickens used in this study were raised in intensive systems and housed in cages equipped with resources for food, water, egg laying, and cage husks. They were fed twice a day, with 100% commercial feed provided for day old chick up to 4 weeks of age, and a mixture of 70% commercial feed and 30% rice bran for 4–12 weeks of age. From 12 to 21 weeks of age, the chickens were given commercial feed and rice bran in a 60:40 ratio, while water was provided ad libitum. Finally, they were vaccinated with the ND vaccine (Medivac ND La Sota, Bandung, Indonesia) on 3 days, 3 weeks, and 3 months of age (Medivac ND Hitchner B1, Bandung, Indonesia).

### Peripheral blood mononuclear cells (PBMCs) isolation

Peripheral blood mononuclear cells were isolated based on the procedures of [9], with some modifications. The blood samples from each group were diluted with phosphate-buffered saline/bovine serum albumin (PBS/BSA) at 1:1. Subsequently, 10 mL of the solution was carefully layered on top of Ficoll in a 15 mL centrifuge tube and centrifuged for 30 min at 350× *g* with no brake. The cells from the serum or separation media were harvested using a pipette and placed in a 15 mL conical centrifuge tube. Phosphate buffered saline/BSA was added to a total volume of 10 mL and centrifuged at 350× *g* for 5 min. The supernatant was removed and a pellet of PBMCs was collected. Finally, the cells were resuspended and adjusted with PBS/BSA to a concentration of 1 × 10^7^ cells/mL.

### Flow cytometry analysis

The percentage of lymphocytes, CD4^+^, and CD8^+^ in the PBMCs were analyzed using flow cytometry [10]. Briefly, 100 mL of the PBMCs were incubated at 4°C for 30 min in a dark place with anti-CD4 and anti-CD8 mouse antibodies which are conjugated with the label PE for CD8 and CD45, as well as FITC for CD4. After incubation, the cells were washed with 2 mL cold PBS/BSA, centrifuged at 350× *g* for 5 min, and the supernatant was then discarded. Following this, the samples were supplemented with 1 mL of PBS/BSA and placed in a flow cytometer cuvette where the total number of cells was counted. The results obtained were subsequently processed using BD cellquest Pro™ (San Jose, USA).

### Statistical analysis

Lymphocytes, CD4^+^, and CD8^+^ populations were analyzed using Minitab, and their means were compared with the t-test.

## Results

**Figure-1:**
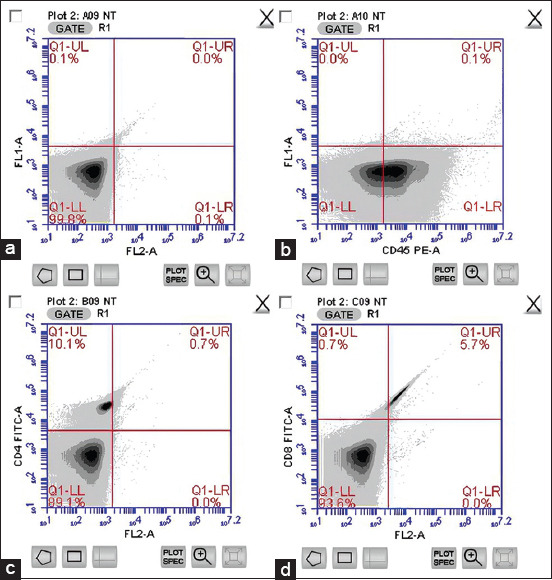
Immunophenotyping of peripheral blood lymphocyte subpopulation. (a) sample control, (b) lymphocytes population, (c) CD4^+^ populations, (d) CD8^+^ populations.

Immunophenotyping of peripheral blood lymphocyte subpopulation was performed by flow cytometry using two different staining panels. Examples of the gating strategies are shown in Figure-1. The results of immunophenotyping in the control sample are presented in Figure-1a. The lymphocyte population, represented by CD45^+^, was located in the lower right position (Figure-1b), while the CD4^+^ and CD8^+^ populations were situated in the upper left (Figure-1c) and upper right (Figure-1d), respectively.

**Figure-2:**
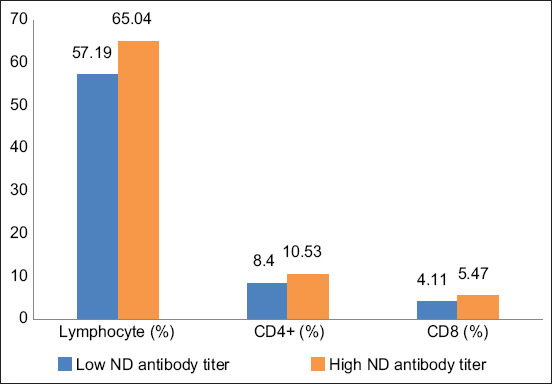
Lymphocytes, CD4^+^ and CD8^+^ population in IPB-D2 chickens.

According to this study, IPB-D2 chicken with high ND antibody titer had higher lymphocytes, CD4^+^, and CD8^+^ than those with low ND antibody titer, as shown in Figure-2. Based on statistical analysis, there were no significant differences in the population of these cells within each group of ND antibody titer levels. The CD4^+^ and CD8^+^ populations in IPB-D2 chickens with high and low ND antibody titers were 1.92 and 2.04, respectively.

## Discussion

Several attempts are being made to understand the immune cells and pathways in local chickens due to the economic importance of these breeds. It is observed that peripheral blood lymphocyte populations are under genetic regulation, hence, providing an important biomarker for evaluating immunocompetence. This study aimed to investigate T lymphocyte subpopulations in IPB-D2 chicken with varying ND antibody titers.

Flow cytometry is a preferred method to phenotype *ex vivo*-derived individual leukocytes. Changes in individual lymphocyte cell concentration are an indicator of pathogen-host interactions. T cells with CD4 and CD8 markers are essential for controlling acute viral infection, while B lymphocytes produce antibodies specific to pathogens [11].

CD45^+^ was discovered on the surface of T and B lymphocytes as well as other leukocytes. It played a specific function by supporting the activation of lymphocytes after binding antigens [12]. The CD45^+^ population in IPB-D2 chickens with high ND antibody titers was higher than those with low ND antibody titers. Furthermore, the increased proliferative activity in peripheral blood lymphocytes showed the strengthening of T and B cells function and increased cellular immunological function [13].

Chickens have CD8^+^ and CD4^+^ T cell subsets, which are parts of cellular immunity. CD8^+^ T cells play a role in cytotoxic responses by killing infected target cells, while CD4^+^ T cells help to eliminate pathogens [14]. These cells are essential for an effective immune response to viral diseases. The majority of CD4^+^ cells are Th and T regulatory lymphocytes [15]. Furthermore, CD4 is a glycoprotein discovered in membranes and functions as a coreceptor in the activation of T-cells expressing restricted Class II MHC. The CD4^+^ marker has been linked to helper T-lymphocytes, and inductively active T-lymphocytes [16].

This study showed that IPB-D2 chickens with high ND antibody titer had higher lymphocytes, CD4^+^, and CD8^+^ than those with low ND antibody titer. The result is consistent with the study by Luo *et al*. [17] that CD4 coded glycoprotein on the surface of Th cell through the interaction with MHC Class II. Furthermore, CD4-activated Th cell and the level of transcription is directly related to the development of T lymphocyte. Mature naïve T cells move from the thymus to peripheral circulation and secondary lymphoid organs, where they are activated in response to antigen exposure [18].

The cytotoxic T-lymphocyte (CTL) response, which is produced by activated CD8^+^ T cells, fights against pathogenic microorganisms. The CD8^+^ CTL response functions by recognizing foreign antigens presented by MHC Class I of infected cells. This recognition triggers the release of perforin and granzyme, causing apoptosis of the infected cells [19].

The CD4^+^ CD8^+^ cell ratio is used to assess the effectiveness of the immune system in various species, including chickens, mice, and humans. Typically, healthy individuals have a CD4^+^:CD8^+^ cell ratio greater than 1, indicating that there are more CD4^+^ cells [20]. This ratio was calculated to determine the relative fluctuation of CD8^+^ cells in comparison to CD4^+^. CD4^+^ and CD8^+^ populations in IPB-D2 chicken with high ND antibody titer were lower than those with low ND antibody titer. Furthermore, a lower ratio of CD4^+^ and CD8^+^ shows that the number of CD4^+^ cells in the circulating T cell population is higher. This is consistent with the category of IPB-D2 chickens with high ND antibody titers, as they have a higher CD4 population. Cluster of differentiation 4^+^ T cells play an important role in adaptive immunity by differentiating and having various functions after being activated by antigens. In addition, it helps in the production of antibodies by B cells [21]. Normal individuals have a CD4^+^ and CD8^+^ ratio of 1–4 [22], and its lower value in chickens has been associated with decreased humoral immunocompetence. IPB-D2 chickens in each category of ND antibody titer have a ratio that is included in normal conditions, indicating that they have good humoral immunity.

## Conclusion

This study concluded that IPB-D2 chickens with high ND antibody titers had higher lymphocyte, CD4^+^, and CD8^+^ populations compared to those with low ND antibody titers. In addition, CD4^+^ and CD8^+^ ratio in IPB-D2 chickens with high ND antibody titers was lower.

## Author’s Contributions

CS, SM, NU, and AG: Designed and supervised the study and revised the manuscript. DL: Sample collection. DL, CS, SM, NU, and AG: Laboratory works and drafted the manuscript. All authors have read, reviewed, and approved the final manuscript.

## References

[1] Lestari D, Murtini S, Ulupi N, Sumantri C (2022). Polymorphism and association of DMA gene with total IgY concentration and ND antibody titer in IPB-D2 chicken line. Trop. Anim. Sci. J.

[2] Biard C, Monceau K, Motreuil S, Moreau J (2015). Interpreting immunological indices:The importance of taking parasite community into account. An example in blackbirds *Turdus merula*. Methods Ecol. Evol..

[3] Kannan T.A, Ramesh G, Ushakumary S, Raj G.D, Viramuthu S (2012). Flow cytometry analysis of CD4 and CD8 T cells in spleen of chicken (Gallus domesticus). Indian J. Vet. Anat..

[4] Yuniwarti E.Y.W, Asmara W, Artama W.T, Tabbu C.R (2012). The effect of virgin coconut oil on lymphocyte and CD4 in chicken vaccinated against Avian Influenza virus. J. Indones. Trop. Anim. Agric.

[5] Kang I (2013). Analysis of T cells using flow cytometry. J. Rheum. Dis..

[6] Fair J.M, Taylor-McCabe K.J, Shou Y, Marrone B.L (2008). Immunophenotyping of chicken peripheral blood lymphocyte subpopulation:Individual variability and repeatability. Vet. Immunol. Immunopathol..

[7] Hofmann T, Schmucker S (2021). Characterization of chicken leukocyte subsets from lymphatic tissue by flow cytometry. Cytometry A.

[8] Lee I. K, Gu M. J, Ko K. H, Bae S, Kim G, Jin G, Kim E. B, Kong Y, Park T. S, Park B, Jung H. J, Han S. H, Y. C (2018). Regulation of CD4^+^CD8^-^CD25^+^ and CD4^+^CD8^+^CD25^+^ T cells by gut microbiota in chicken. Scientific Reports.

[9] Azizi M.R, Asli E, Behroozikhah A.M, Khalesi B (2021). Flow cytometry evaluation of CD4^+^ and CD8^+^ T-ce;immune response in SPF chickens induced by fowlpox vaccine. Arch. Razi. Inst..

[10] Pinca S, Djati M.S, Rifa'I M (2013). Analisis mobilisasi sel T CD4+dan CD^8^+pada timus ayam pedaging pasca infeksi *Salmonella tyhimurium* dan pemberian *simplisi*a *Polyscias obtusia*. J. Penelitian Univ. Brawijaya.

[11] De Boever S, Croubles S, Demeyere K, Lambrecht B, De Backer P, Meyer E (2010). Flow cytometric differentiation of avian leukocytes and analysis of their intracellular cytokine expression. Avian Pathol..

[12] Popovic M, Balenovic M, Kabalin A.E, Savic V, Vitjtiuk N, Vlahavic K, Valpotic I (2010). Evaluation of CD45^+^ cells kinetics in the blood of fattening chickens immunized with live or inactivated Newcastle disease vaccine. Vet. Arch.

[13] Song B, Tang D, Yan S, Fan H, Li G, Shahid M.S, Mahmood T, Guo Y (2021). Effect age of on immune function in broiler chickens. J. Anim. Sci. Biotechnol..

[14] Alvarez K.L.F, Poma-Acevedo A, Sanchez M.F, Fernandez-Diaz M (2020). An EdU-based flow cytometry assay to evaluate chicken T lymphocyte proliferation. BMC Vet. Res.

[15] Astwa N.N, Oka I.D.B.M (2016). Lymphocytes subpopulation in peripheral blood and spleen of village chickens recognized by monoclonal antibodies. J. Vet..

[16] Komich V.T, Dyhliuk N.V, Mazurkevych T.A, Guralska S.V, Usenko S.I (2021). Content and location of lymphocyte subpopulation with markers CD4^+^, CD8^+^ and CD20^+^ in the esophageal tonsil of chickens and the Meckel diverticulum of ducks. Regul. Mech. Biosyst..

[17] Luo J, Yu Y, Zhang H, Tian F, Chang S, Cheng H.H, Song J (2010). Down-regulation of promoter methylation level of CD4 gene after MDV infection in MD-susceptible chicken line. BMC Proc.

[18] Wang C.J, Yu S, Ao-Ri-Ge-Le Jia, D, Yoa H.Q, Zhao H.P, Lillehoj H.S, Si-Mu-Ji-De, Postnikoff A.C.L, Xu S (2012). Regulation of T lymphocyte subpopulations in specific pathogen-free chickens following experimental fowl adenovirus VIII infections. Braz. J. Microbiol.

[19] Umthong S, Duan J.R, Cheng H.H (2020). Depletion of CD8ab^+^ T cells in chickens demonstrates their involvement in protective immunity towards Marek's disease with respect to tumor incidence and vaccinal protection. Vaccine.

[20] Mortada M, Cosby D.E, Akerele G, Ramadan N, Oxford J, Shanmugasundaram R, Ng T.T, Selvaraj R.K (2021). Characterizing the immune response of chickens to *Camphylobacter jejuni* (strain A74C). PLoS One.

[21] Rushdi M.N, Pan V, Li K, Choi H, Travaglino S, Hong J, Griffitts F, Agnihorti P, Mariuzza R.A, Ke Y, Cheng S (2021). Cooperative binding of T cell receptor and CD4 to peptide-MHC enhances antigen sensitivity. Nat. Commun..

[22] Febrianty H, Dhatu M.S (2015). Modulasi sel T CD4^+^ dan CD8^+^ pada spleen ayam arab putih (Gallus turcicus) dengan ransum yang mengandung daun papaya *(Carica papaya* L.). J. Biotropika.

